# Ameliorating orthodontic relapse using laser bio-stimulation and mesenchymal stem cells in rats

**DOI:** 10.1016/j.jgeb.2023.100331

**Published:** 2024-01-22

**Authors:** Samer S. Othman, Ali Saafan, Mohammad M.F. Al-Halbosiy, Iman Fathy, Mohamed Khursheed Alam, Amr R. El-Beialy, Hanady S. Al-Shmgani, Ghassan M. Sulaiman

**Affiliations:** a Department of Clinical Sciences, College of Dentistry, Ibn Sina University of Medical and Pharmaceutical Sciences, Baghdad, Iraq; b Department of Medical Applications of Laser, National Institute for Laser Enhanced Sciences, Cairo University, Cairo, Egypt; c Biotechnology Research Center, Al-Nahrain University, Baghdad, Iraq; d Department of Oral Biology, Faculty of Dentistry, Ain Shams University, Cairo, Egypt; e Professor of Orthodontics, Department of Preventive Dentistry, College of Dentistry, Jouf University, Sakakah, Saudi Arabia; f Department of Orthodontics and Dentofacial Orthopedics, Faculty of Dentistry, Cairo University, Cairo, Egypt; g Department of Biology, College of Education for Pure Sciences, Ibn Al-Haitham University of Baghdad, Baghdad 10066, Iraq; h Division of Biotechnology, Department of Applied Sciences, University of Technology, Baghdad 10066, Iraq

**Keywords:** Orthodontic relapse, Mesenchyme stem cells, Low-level laser treatment, Laser irradiation, Hetero-formed periodontal ligament

## Abstract

**Background:**

Orthodontic relapse is a frequent problem that many patients experience. Although orthodontic therapy has advanced, recurrence rates can still reach 90%. We undertook a study to look at the possibilities of laser bio-stimulation and stem cells because they have showed promising outcomes in lowering recurrence rates.

**Objectives:**

Our objective was to analyze the effects of Low-level laser therapy (LLLT) and Mesenchymal stem cells (MSC) alone and collectively on the rate of orthodontic relapse in rats radiographically and histologically.

**Methods:**

Rat maxillary central incisors were moved distally for two weeks. One week later, the incisors were retained. Animals (n = 40) were split into four groups. Control group (C); laser treatment Group (L), Bone marrow mesenchymal stem cells Group (BMSCs) and combination of Stem cells and laser-irradiation group (BMSCs-L). Removed retainer permitted relapse. Before stem cell application or laser irradiation, each animal underwent two CBCT scans. Rat maxillae were stained with Hx&E, Masson trichrome, and tartrate-resistant acid phosphatase antibody for histology, histochemistry, and immunohistochemistry.

**Results and conclusions:**

LLLT could reduce the relapse tendency, as shown by increased bone density and enhanced remodeling of hetero-formed periodontal ligament (PDL). Furthermore, the transfer of BMMSCs on the pressure side had positive effects on PDL remodeling and decreased, but did not inhibit, the relapse rate. Finally, the synergistic effects of the application of LLLT and BMMSC were better than the control but still moderate and long-lasting.

**Clinical Significance:**

Based on the improved relapse rate as proven in the present study, the Application of both LLLT and stem cells can be adopted to reduce the relapse tendency either lonely or collectively.

## 1 Introduction and background

Multi factors cause orthodontic relapse. Redevelopment of the hetero-formed periodontal ligament (PDL) and bone remodeling surrounding moved teeth may explain slow relapse after orthodontic tooth movement (OTM).^1^ In humans, most relapses occur during the first year after retention. Still, in rats, the mean relapse occurred one day after the expulsion of orthodontic pressures, with the velocity of relapse declining over time.^2^ These findings were explained at the cellular level,^3^ where pharmacologic treatments decreased recurrence in animal models. Bisphosphonate,^4^ osteoprotegerin^5^ simvastatin,^6^ relaxin,^7^ and BMPs^8^. Relapse was minimized by modifying dent-al supporting tissues.

Mesenchymal stem cells (MSCs) are multipotent postnatal stem cells that can be self-renewed and transformed into different types of cells that are fundamental for tissue recovery.^9^ Recent studies documented the role of MSCs in reducing inflammation and generating cytokines associated with tissue regeneration. Exogenous MSCs might promote tissue formation through their differentiation or activating endogenous progenitor cells when exposed to an inflamed microenvironment.^10^

Low-level laser treatment (LLLT) has been widely used in dentistry due to its ability to cause a rise in cell proliferation rates, such as fibroblasts, endothelial cells, osteoblasts, epithelial cells, and lymphocytes.^11^ A beneficial effect of LLLT on bone marrow and adipose tissue stem cells has been documented in the literature on mesenchymal stem cell proliferation.^12^ Accordingly, LLLT bio stimulation might enhance bone redesigning, with expanded collagen union, bone development and mineralization, cell expansion and separation, and angiogenesis.^13^ Notwithstanding the common finding of improved OTM, a few examinations reported that a large portion of laser biomodulation hindered OTM.^14^ It was hypothesized that LLLT might reduce relapse due to accelerated bone regeneration.^15^

Up-to-date, insufficient knowledge is available on the effect of laser biostimulation and mesenchymal stem cells on the rate of orthodontic relapse. Hence, the presented study aimed to analyze the effects of LLLT and MSCs alone and collectively on the rate of orthodontic relapse in rats radiographically and histologically.

## 2 Methods

### 2.1 Animals and experimental procedure

The experimental animal protocol was approved by the Institutional Animal Care and Use Committee (CU-IACUC) (Approval No. CUIF6518). In this study, a total of 40 male Wistar rats weighing (120–160 g) were included.

Rat maxillary central incisors endured a distal orthodontic movement for two weeks using elastic separator rings. This procedure was performed under general anesthesia with Ketalar 10 mg/ml and Zoletil (0.25 mg/kg). After stopping orthodontic tooth movement, the distally moved central incisors were temporarily fixed using a wire-resin splint retainer for one week.^15^ At this point, the animals were randomly divided into four groups, each group consisting of 10 rats:

Group (C): Control group. Group (L): The low-level laser was used on the mesial aspect of the root of the central maxillary incisors. Group (MSCs): a total of 5x10^4^ Bone marrow mesenchymal stem cells were used, half density on the mesial aspect of the root of each incisor. Group (MSCs-L): Combined group (MSCs-L) with both MSCs and LLLT application.

The retainer was removed afterward to allow for relapse movement. The animals of each group (2 rats from each group each time) were sacrificed by an overdose of Zoletil 50 (0.25 mg/kg, IM) at four days, 1, 2, 3, and 4 weeks after retention removal.

### 2.2 Sample preparation

#### 2.2.1 Groups (L) & (MSCs-L)

This investigation employed a gallium-aluminum-arsenide (GaAlAs) diode laser device with 808 nm wavelength and 100 mW for 180 sec with 18 J output. A 0.35 cm-radius, 0.385 cm2 probe tip delivered the laser beam. 46.8 J/cm2 were applied. The probe lightly touched the labial gingiva over each central incisor's root. From the day orthodontic tooth movement stopped till euthanasia, LLLT was performed every two days.

#### 2.2.2 Groups (MSCs) & (MSCs-L)

The technique for preparing mesenchymal stem cells was carried out in accordance with Rochefort et al.^16^^.^ After isolation and passage; adherent cells were examined by FACS Canto II using a 488 nm Argon laser. Cells were treated at 4° C for 60 min with monoclonal antibodies against rat CD45 (Clone REA504), rat CD90 (Clone REA838), and rat CD29 (Clone REA683). Controls were isotype-identical antibodies. 10,000 FACS Canto II Flow Cytometer events were collected and analyzed with BDFACS DivaTM v6.1.3 software.

BMMSCs were transferred to the mesial PDL of rats' upper central incisors under general anesthesia. The injection site was determined using Cone Beam Computed Tomography (CBCT) scans performed before the MSCs transfer. Non-traumatic injections were performed.^17^

#### 2.2.3 Cone beam computed tomography (CBCT)

Before stem cell application or laser irradiation (T1) and before euthanasia (T2), each animal underwent two CBCT scans (T2). Planmeca ProFaceTM 3D CBCT equipment, 90 kV, 12.5 mA for 12 s. Using the rat's forehead as a reference plane, the distance between the orthodontic tooth movements (OTM) was measured. The average crown-to-root distance was determined. Relapse distance is the difference between T1′s OTM and T2′s remaining space.

The bone mineral density of the area between the mesial aspects of central incisors and the area distal to each central incisor was measured. The reference plane determined each area's vertical portion. Hounsfield units measured coronal and axial bone density. Bone density was evaluated using the forehead as a reference plane to discover group differences after relapse. After two weeks, an examiner repeated all measures twice to determine technique error.

#### 2.2.4 Histological preparation

The maxillae of the rats were excised, given code numbers, fixed with 10 % neutral formaldehyde. After 72 h, the samples were demineralized in a solution having equal parts of 50 % formic acid and 20 % sodium citrate for 45 days. The bodies of the rats were appropriately removed from the incinerator of the Ain-Shams hospital.

Specimens were processed into paraffin blocks and microtome nonconsecutive sections (5 mm apart) were cut from paraffin blocks at 50 µm (Leica RM2125RT, Nussloch, Germany). Bindhu et al. stained cut sections with H&E and Masson trichrome.^18^ Coverslip portions were taken with a microscope camera (Nikon Eclipse E600, Japan). To ensure unbiased evaluation, the same light settings were used for photographing and histochemical examination.

#### 2.2.5 Immunohistochemical staining

After de-paraffinization and rehydration of sections, endogenous peroxidase activity was suppressed by incubating the specimen in 3 % H2O2 in methanol at 23oC for 15 min. The polyclonal primary tartrate-resistant acid phosphatase (TRAP) was applied for 60 min at 23° C. The antibody's species reactivity to humans and rats distinguishes it. The slices were then incubated for 30 min at room temperature with a secondary biotinylated antibody. The slides were then incubated at room temperature for 5 min with BioCare diaminobenzidine with chromogen. The sections were counterstained for 2 min with hematoxylin, washed in distilled water, dehydrated in graded alcohol, and cleaned in xylene. Finally, specimens were mounted on positive slides with aqueous mounting media before being viewed under a light microscope. Positive reactions seemed brown in hue. This antibody's cellular location is cytoplasmic. Blue was identified as the negative reaction. Negative controls were carried out by omitting primary antibodies and replacing them with nonimmune serum.^19^

#### 2.2.6 Histomorphometry analysis

The photographs were taken with a Leica DMi50 light microscope and Leica ICC50 digital camera. Morphometric analysis was done using 'ImageJ' software (version 1.48v National Institute of Health, Bethesda, Maryland, USA). Each sample includes five random nonoverlapping fields from each slide at 400x. A single-blind examiner detected osteoclasts on the PDL's pressure and tension sides. Large multinucleated cells with dark cytoplasm were categorized as osteoclasts. Microsoft Excel tabulated the data.

### 2.3 Statistical analysis

IBM® SPSS® Statistics Version 25 for Windows was employed for the statistical analysis. For each subgroup, the mean and standard deviation were computed. The Shapiro-Wilk test for testing normality found a normal distribution between the values of each group. Using the Levene test, a homogeneity test found that all variables have a homogenous distribution. Therefore, a one-way ANOVA test (with a significance level of P 0.05) was conducted between the variables.

## 3 Results

1. MSCs **characterization**

BMMSCs have 98.5 % and 98.6 % positive CD29 and CD90. Only 0.7 % of hematopoietic cells expressed CD45-label, indicating that the separated BMMSCs are homologous and pure mesenchymal stem cells.

**Fig. 1:**
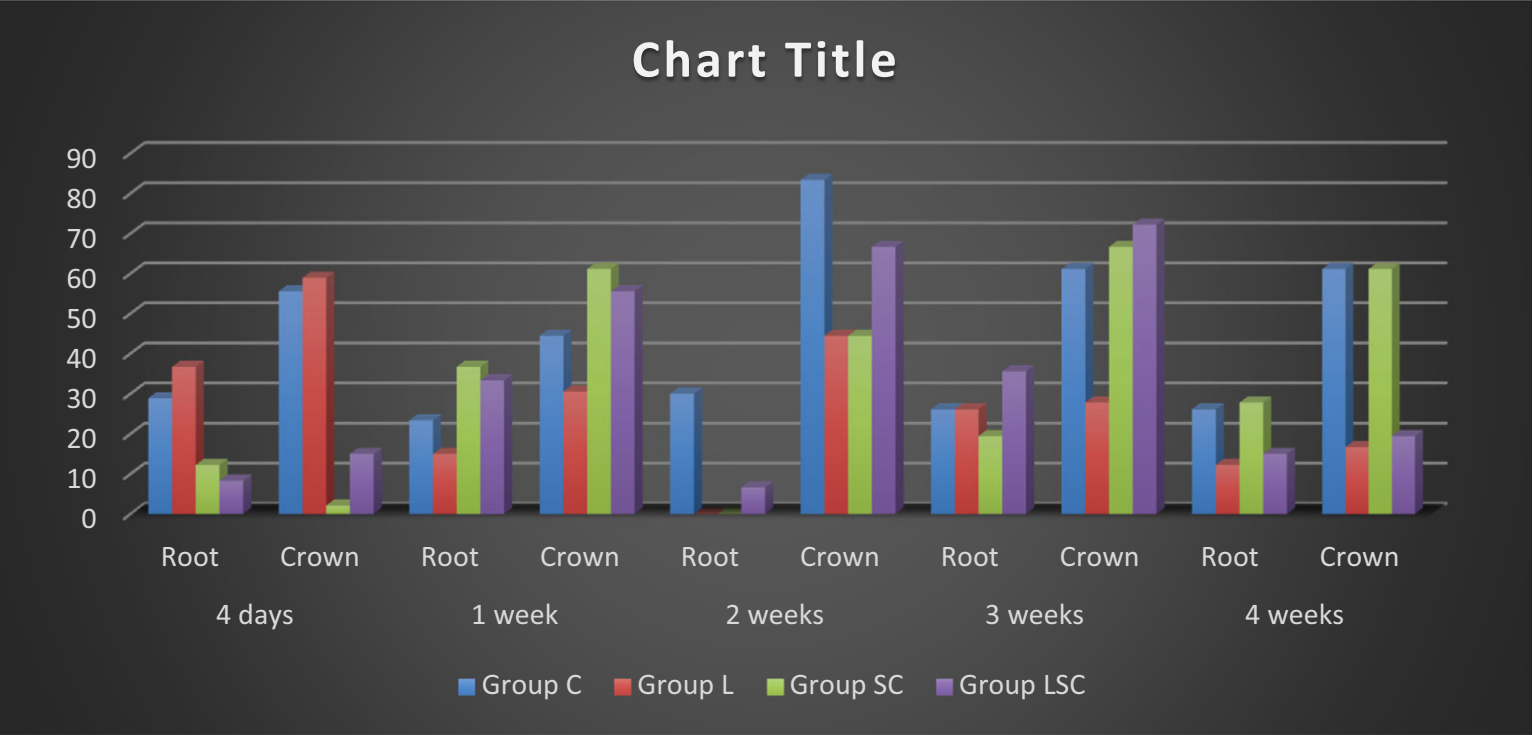
bar chart representing relapse rate of crowns and roots in different subgroups.
b. Crown area: The experimental groups differed significantly after 4 and 1 weeks. The L group had the most remarkable 4-day relapse rate, which reduced over time. MSC had the most special 1-week relapse rate. 2-, 3-, and 4-week testing periods showed no statistically significant differences. (Fig. 1)
ii. **Bone density:**
a. Mesial aspect: The L group had the highest bone density for four days and one week, with a significant difference for one week. MSCs had the highest bone density at 2 and 4 weeks, with a substantial difference from L at three weeks. The C group exhibited the highest bone density at three weeks, while MSCs-L had moderate values. (Fig. 2)

2. **Cone Beam Computed Tomography (CBCT**
i. Relapse rate:
a. Root area: The C group had the most remarkable relapse rate at 2 points, the only statistically significant difference. The L group had the highest relapse rate for four days and the lowest for two weeks, while the MSC group had the highest for one week. C and MSC-L were moderate. (Fig. 1)

**Fig. 2:**
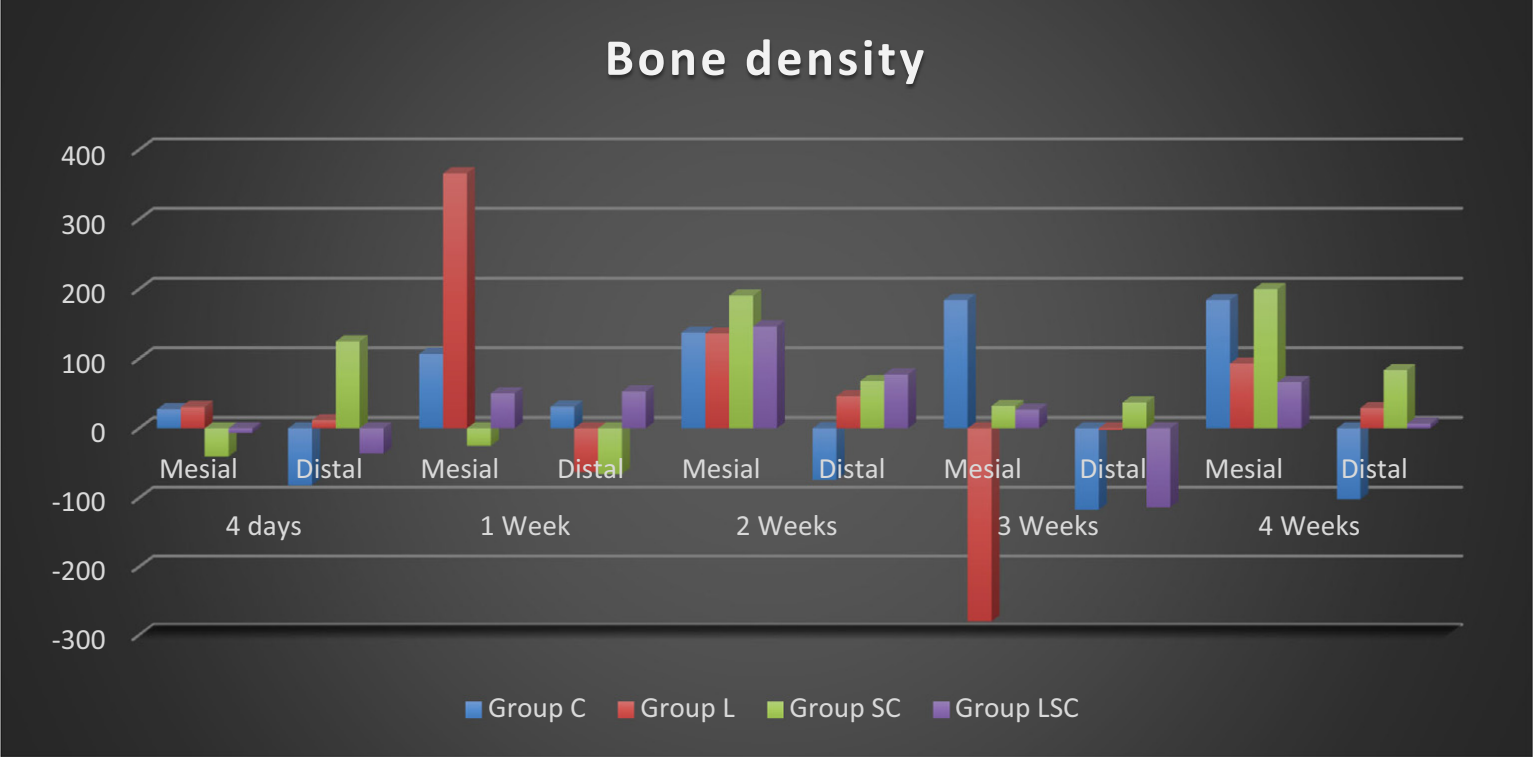
bar chart representing the bone density of crowns and roots in different subgroups.
b. Distal aspect: The MSCs-L group had the highest results at four days and one week, whereas the L group had the lowest. MSC showed the highest bone density values at two, three, and four weeks, while C showed the most inferior. The L group's bone density was considered moderate throughout this experiment. (Fig. 2)
3. **Histological Analysis (Hematoxylin and Eosin results and Masson trichrome results):**
i. After four days: On the mesial side, the MSC Group showed active resorption, and the periodontal ligament had blocked blood vessels and hyalinization. Alveolar bone margins displayed osteoclast-like scalloping. The C group had the most ordered periodontal tissue structure, proper blood vessel distribution, well-organized collagen fibers, and extensive interstitial tissue. Similar to the L group. Active osteogenesis was seen as plump, healthy osteoblasts in the distal wall of L and MSC groups. C and MSCs-L revealed typical periodontal ligament architecture and vasculature. (Fig. 3-A).

**Fig. 3:**
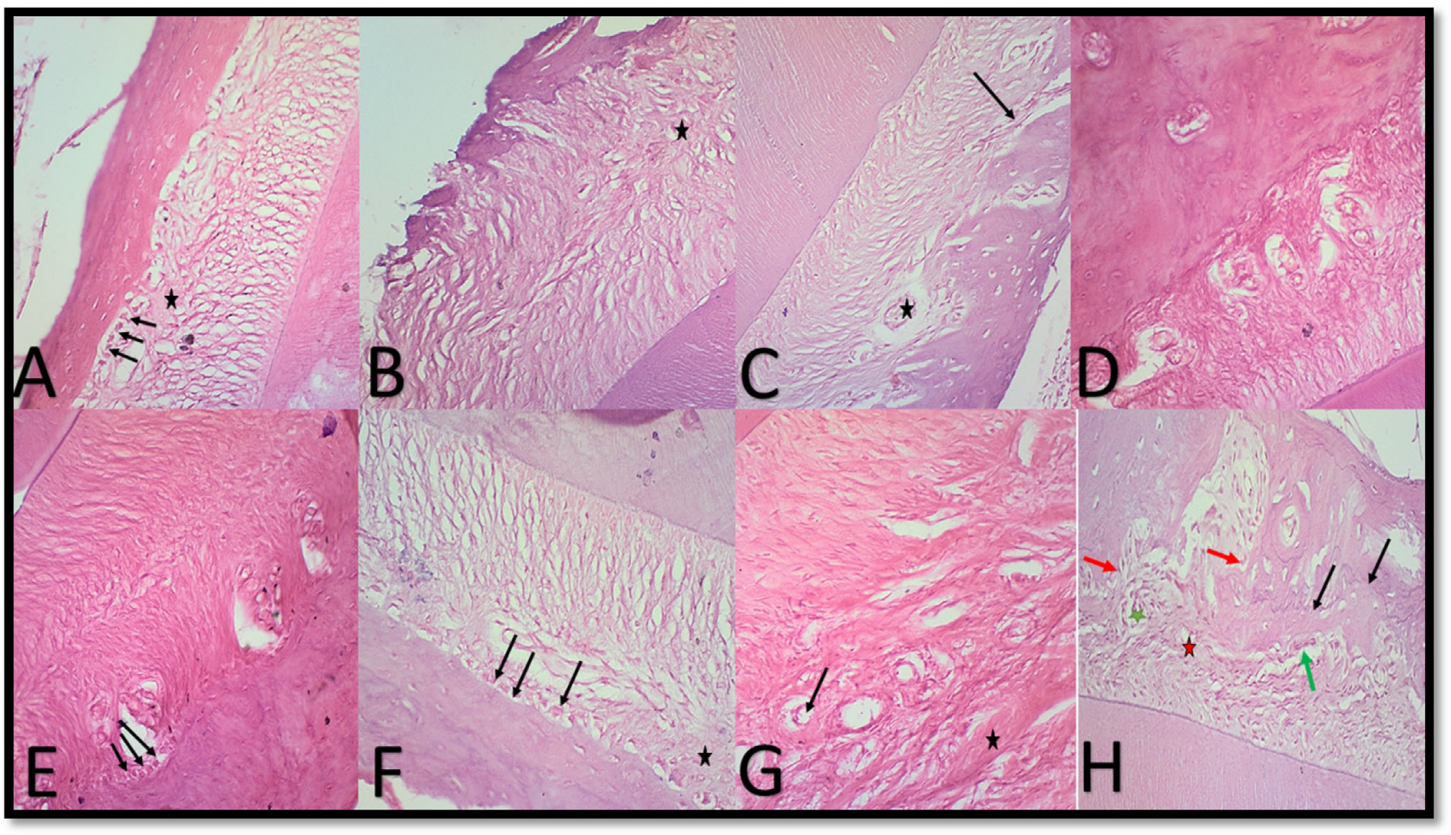
plate showing photomicrographs of different subgroups. A: mesial wall in MSCs group after 4 days showing resorptive findings as osteoclast like cells along scalloped margin of the alveolar bone (arrow) and disrupted PDL arrangement with areas of hyalinization (star);B: distal wall in MSCs group after 1 week showing reorganizing of PDL fibers, interstitial tissue (star); C: distal wall in MSCs-L group after 1 week showing viable osteocytes in lacunae, patent Zucker Kandle and Hershfield canal (arrow) and interstitial tissue (star);D: mesial wall in group L after 2 weeks showing interstitial tissue in PDL and almost smooth margin of alveolar bone that contains viable osteocytes in lacunae;E: distal wall in MSCs-L group after 2 weeks showing more organized Periodontal tissue in both bone and tooth sides compared to mesial wall with smooth alveolar bone margin and a well-organized mineralizing front as osteoblast like cells are noticed (arrows); F: mesial wall in group L after 2 weeks showing resorptive findings as osteoclast like cells along scalloped margin of alveolar bone (arrows) and disrupted PDL arrangement with areas of hyalinisation (star);G: distal wall in MSCs group after 2 weeks showing abundant interstitial tissue with wide patent blood vessels (arrows) with persistant areas of hyalinization (star);H: mesial wall in MSCS-L group after 4 weeks showing multiple reversal lines (black arrows), areas of bone formation with osteoblast like cells (green arrows) and areas of bone resorption with osteoclast like cells (red arrows), PDL shows areas of hyalinisation (red star) beside interstitial tissue (green star).[H&E X200]. (For interpretation of the references to colour in this figure legend, the reader is referred to the web version of this article.)
ii. After one week: After one week, the C and MSC groups' distal and mesial walls reorganized PDL fibers, interstitial tissue, patent Hirschfield canals, and viable osteocytes in the mesial wall of the lacunae. The L group showed a well-organized mineralization front mesial on the alveolar bone proper margin with plump osteoblast-like cells, abundant vasculature, and organized new collagen fibers in PDL with abundant interstitial tissue. The distal alveolar bone proper margin showed giant osteoclast-like cells. Distal and mesial MSCs-L wall fibers, interstitial tissue, patent Zucker Kandle, and Hirschfield canal reorganized after one week. (Fig. 3-B & C; Fig. 4-A, B&C)

**Fig. 4:**
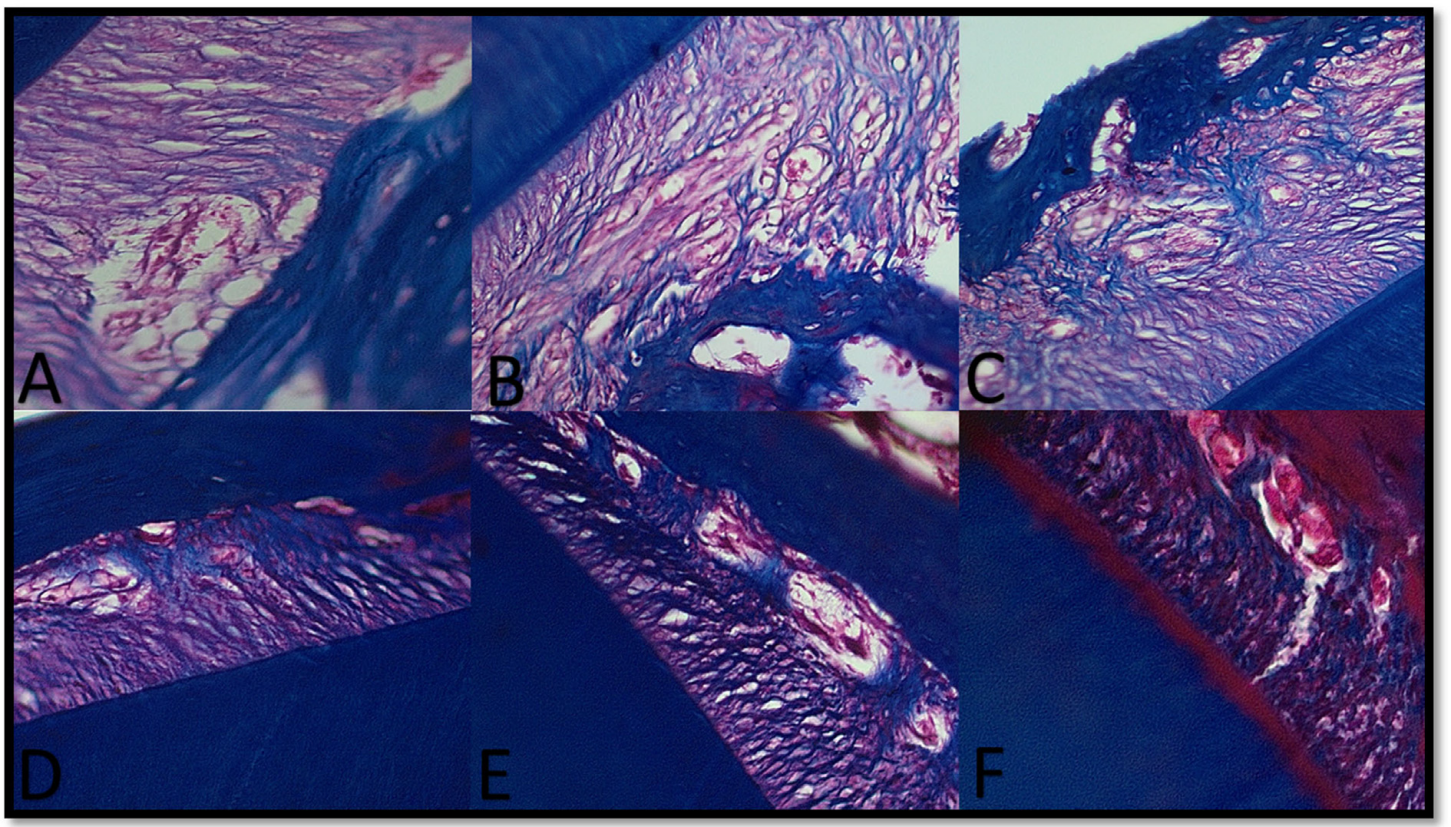
plate showing photomicrographs of different subgroups. A: mesial wall in group L after one week showing organized new collagen fibers in PDL with abundant interstitial tissue and patent b.v.(stars); B: distal and mesial walls in MSCs-L group after one week showing reorganizing of PDL fibers with wide interstitial tissue (stars),C: MSCs-L group after 1-week patent Zucker Kandle and Hershfield canal (arrow)and interstitial tissue (stars);D: distal wall in MSCs group after two weeks showing interstitial tissue (star) in well-organized PDL of mixed old and new collagen and almost smooth margin of alveolar bone; E: mesial wall in L group after three weeks showing less organized Periodontal tissue in the bone side compared to the distal wall with smooth alveolar bone margin and some giant cells with vascularized interstitial tissue (arrows); F: distal wall in MSCs -L group after four weeks showing a stable arrangement of old and new collagen fibers in Pdl with persistent wide vascularized interstitial tissue in the bone side with a smooth margin.[Massson trichrome X200].
iii. After two weeks: The mesial side of Group C demonstrated bone growth and PDL organization after two weeks, but the distal wall showed resorption and PDL disruption. After two weeks, the mesial wall of the L group revealed resorptive osteoclast-like cells along the scalloped alveolar bone and disrupted the PDL arrangement with hyalinized regions. After two weeks, MSCs showed extensive interstitial tissue, large patent blood arteries, and hyalinization. MSCs-L had similar but less evident results to L and MSCs. (Fig. 3-D, E, F & G; Fig. 4-D)
iv. After three weeks: After two weeks, Group C's mesial side showed bone development and PDL organization, but the distal wall showed resorption and PDL disruption. After two weeks, the mesial wall of the L group displayed resorptive osteoclast-like cells and disturbed the PDL arrangement with hyalinized areas. MSCs showed interstitial tissue, large patent blood vessels, and hyalinization after two weeks. MSCs-L demonstrated similar effects to L and MSCs. (Fig. 4-E)
v. After four weeks: All groups showed indications of healing and restructuring of PDL fibers after four weeks, coupled with plentiful vasculature and patent Zucker Kandle and Hirschfield canals. The earlier discovery was more evident in the mesial C and MSC groups, while the distal C group showed evidence of PDL disruption and dispersed large bone-resorbing cells.

PDL reveals areas of hyalinization near interstitial tissue on the mesial side of the MSCs-L group at four weeks, which has many reversal lines, osteoblast-like cells, and osteoclast-like cells. Active osteoblasts with good vasculature were detected in the L group distal wall. (Fig. 3-H).

4. Immunohistochemical analysis (Anti- Tartrate Resistant Acid Phosphatase -TRAP results):
i. After four days: On the mesial side: Groups L and MSCs showed the most intense positive reaction where the alveolar bone proper was seen to be rimed by positively stained giant cells mostly existing in lacunae. Group C had a minor positive response with almost no positive cells in some sections.

**Fig. 5:**
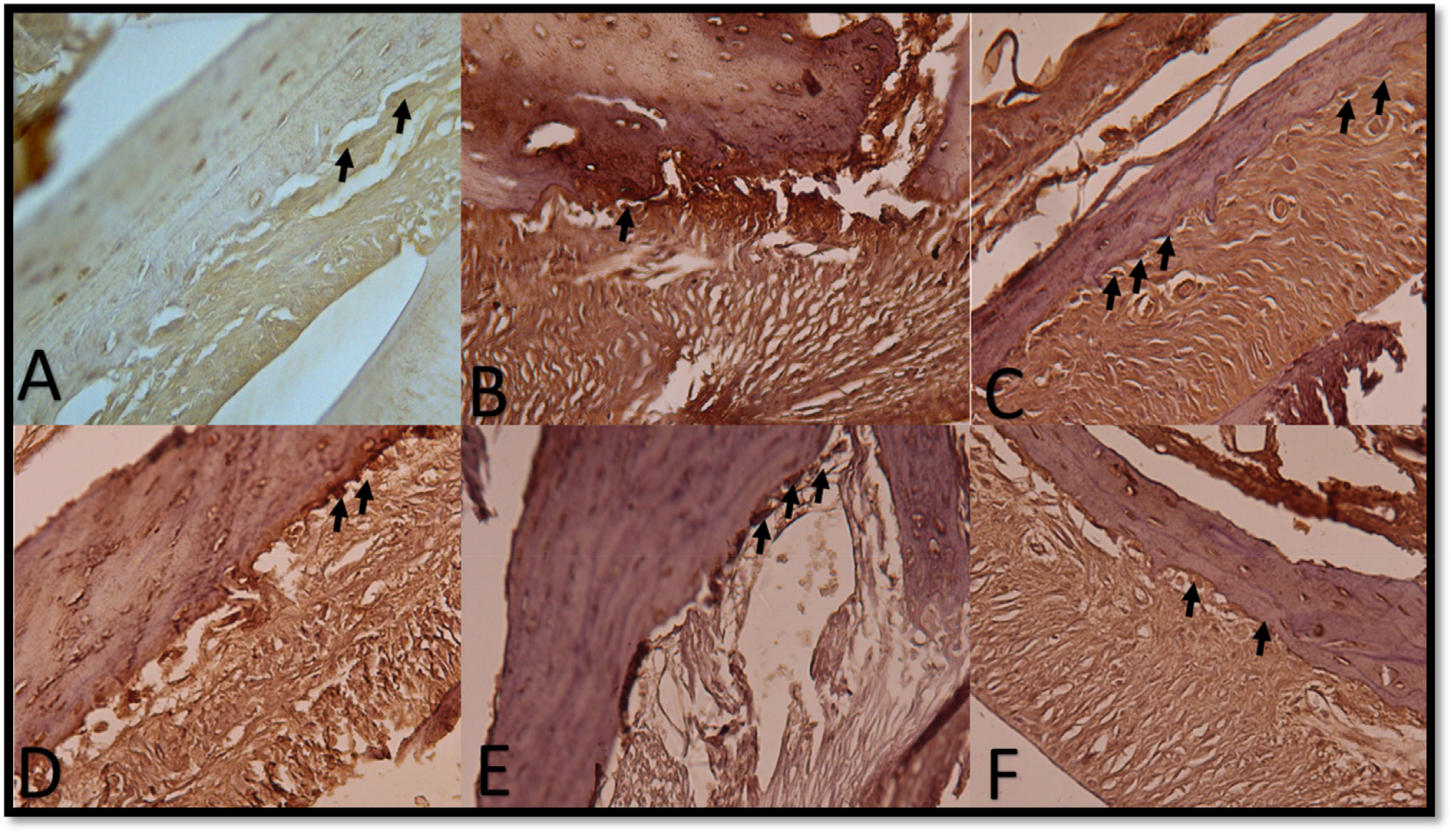
plate showing photomicrographs of different subgroups. A: mesial wall in the C group after four days showing few positive cells along the alveolar bone (arrows); B: mesial wall in the MSC group after one week showing a decrease in positively stained cells (arrow) near a patent Zucker Kandle and Hershfield canal; C: mesial wall in the MSC group after two weeks showing giant giant giant positive stained cells along the scalloped margin of the the the alveolar bone; D: mesial wall in the the the MSCS-L group after two weeks showing less density of the the the positively stained cells population (arrows); E: mesial wall in the the the MSCS-L group after three weeks showing the highest detected population of positive cells. (arrows); F: mesial wall in the MSC group after four weeks of a low density of positively stained cells. [anti- Tartrate Resistant Acid Phosphatase X400].

**Fig. 6:**
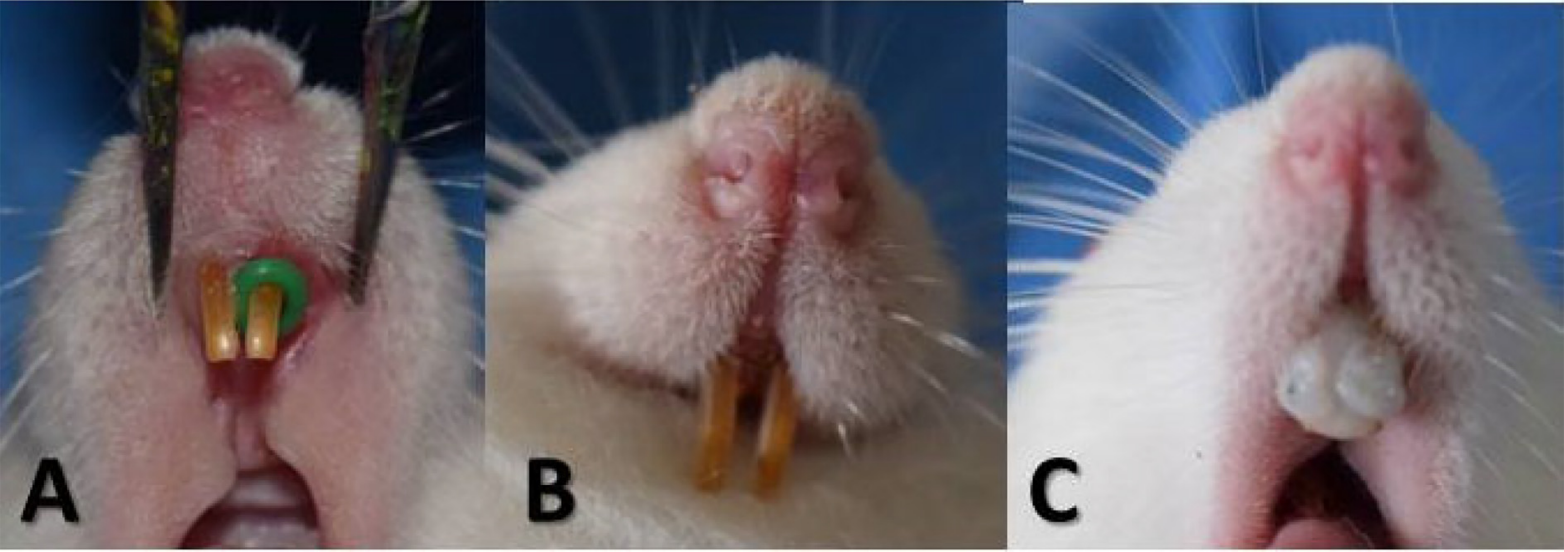
Tooth movement and retention appliance: A. elastomeric ring applied on the central incisor, B. tooth movement after 2 weeks, C. wire splinteed retainer for 1 week.

On the distal side, L and MSC groups showed a very slight positive reaction. (Fig. 5-A).

ii. After one week: Mesial & distal walls in the MSC group after one week showed the most intense positive reaction and giant cell count in this duration, while a mesial wall in the L group showed less positive response than noticed in 4 days. After one week, the distal wall in Group C showed a smooth alveolar bone margin with a low positive reaction, and a well-organized mineralizing front as osteoblast-like cells was observed. (Fig. 5-B)
iii. After two weeks: The mesial wall of the L group after two weeks showed giant positive stained cells along the scalloped margin of the alveolar bone and the distorted PDL arrangement. MSCs group after two weeks showed a decrease in detected positive cells. (Fig. 5-C & D)
iv. After three weeks: The highest detected population of positive cells was found in the proper mesial alveolar bone sections in the MSC-L group, while the C group showed the lowest density of positive cells with a smooth alveolar bone margin. (Fig. 5-E)
v. After four weeks: The mesial side of the MSCs-L group after four weeks showed the most intense positive reaction where alveolar bone proper was seen to be rimmed by positively stained giant cells, in contrast to the low density of positively stained cells at the same position in the L group. In contrast, a small number of positive cells were observed in the distal wall in the L group to active osteoblasts. (Fig. 5-F)
6. The statistical analysis of the morphometric results (Osteoclast cell count):

The C, L, and MSC groups showed a statistically significant difference in osteoclast numbers between mesial and distal aspects at four days, one-week, and two weeks intervals, with no significant differences at other intervals of our study. However, the MSCs-L group revealed a significant difference in the 1-, 3-, and 4-week intervals, with no significant differences in the 4-day and 2-week periods.(Table. 1, Fig. 6).

**Table 1:** Descriptive statistics of intergroup comparison of osteoclasts count at each observation time point.

	**4 days**		**1 week**		**2 weeks**		**3 weeks**		**4 weeks**		**P Value**	
	**Mesial** **Mean ± SD**	**Distal Mean ± SD**	**Mesial** **Mean ± SD**	**Distal Mean ± SD**	**Mesial** **Mean ± SD**	**Distal Mean ± SD**	**Mesial** **Mean ± SD**	**Distal Mean ± SD**	**Mesial** **Mean ± SD**	**Distal Mean ± SD**	**Mesial**	**Distal**
**Group C**	**13 ±** **0.89**	**11 ±** **1.79**	**12 ±** **0.89**	**10 ±** **1.79**	**13 ±** **0.89**	**12 ±** **0.89**	**11 ±** **0.89**	**10 ±** **0.89**	**12 ±** **1.79**	**11 ±** **1.79**	0.027*	**0.146**
	**c** **A**	**c**	**d** **AB**	**c**	**c** **A**	**c**	**d** **B**	**e**	**e** **AB**	**b**		
**Group L**	**24 ±** **0.89**	**18 ±** **1.79**	**23 ±** **1.79**	**16 ±** **0.89**	**24 ±** **3.58**	**18 ±** **0.89**	**19 ±** **0.89**	**15 ±** **0.89**	**14 ±** **0.89**	**13 ±** **0.89**	0.000*	0.000*
	**a** **A**	**a** **A**	**b** **A**	**b** **B**	**a** **A**	**a** **A**	**bc** **B**	**bc** **B**	**de** **C**	**b** **C**		
**Group SC25**	**15 ±** **1.79**	**12 ±** **0.89**	**27 ±** **0.89**	**20 ±** **1.79**	**19 ±** **1.79**	**16 ±** **1.79**	**20 ±** **2.68**	**18 ±** **0.89**	**16 ±** **1.79**	**14 ±** **3.58**	0.000*	0.000*
	**bc** **D**	**c** **D**	**a** **A**	**a** **A**	**b** **BC**	**ab** **BC**	**b** **B**	**ab** **AB**	**cd** **CD**	**b** **CD**		
**Group SC50**	**23 ±** **1.79**	**16 ±** **0.89**	**23 ±** **0.89**	**15 ±** **0.89**	**17 ±** **1.79**	**12 ±** **1.79**	**18 ±** **1.79**	**14 ±** **2.68**	**18 ±** **2.68**	**13 ±** **1.79**	0.000*	0.005*
	**a** **A**	**ab** **A**	**b** **A**	**b** **AB**	**b** **B**	**c** **C**	**bc** **B**	**c** **ABC**	**bc** **B**	**b** **BC**		
**Group LSC25**	**18 ±** **2.68**	**15 ±** **1.79**	**18 ±** **1.79**	**14 ±** **3.58**	**16 ±** **1.79**	**13 ±** **3.58**	**25 ±** **0.89**	**20 ±** **4.47**	**24 ±** **1.79**	**19 ±** **1.79**	0.000*	0.002*
	**b** **B**	**b** **ABC**	**c** **B**	**b** **BC**	**bc** **B**	**bc** **C**	**a** **A**	**a** **A**	**a** **A**	**a** **AB**		
**Group LSC50**	**18 ±** **1.79**	**17 ±** **0.89**	**18 ±** **0.89**	**17 ±** **1.79**	**18 ±** **1.79**	**16 ±** **2.68**	**16 ±** **3.58**	**12 ±** **0.89**	**20 ±** **0.89**	**14 ±** **1.79**	0.043*	0.000*
	**b** **AB**	**ab** **A**	**c** **AB**	**ab** **A**	**b** **AB**	**ab** **AB**	**c** **B**	**cd** **C**	**b** **A**	**b** **BC**		
**P value**	0.000*	0.000*	0.000*	0.000*	0.000*	0.000*	0.000*	0.000*	0.000*	0.000*		
● Indicates the mean difference is statistically significant at the 0.05 level. ● Different small litter indicates statistically significant difference in the same column. (p-value ≤ 0.05). ● Different capital litter indicates statistically significant difference in the same rows. (p-value ≤ 0.05).

Throughout the experiment periods, the number of osteoclasts in the mesial aspect was higher than its number in the distal aspect; also, the osteoclast count in the compression side showed elevated numbers during the four days, week, and two weeks intervals in the L and MSC groups. In contrast, the MSCs-L group showed elevated osteoclast numbers in the 3- and 4-week periods. Group C mirrored the lowest osteoclast cell counts during all experiment periods on both aspects.

## 4 Discussion

In this study, each animal received 18 J/session with 180 s of exposure and 46.8 J/cm2 total energy density. Comparative investigations 15, 20 employed 20–23 J/cm2 energy density. In other research, laser irradiation at 20–25 J/cm2 accelerated bone remodeling in rat bone defects ^20^^.^ Bardach and Hadad^21^ found that recommending lower energy densities is misleading. Instead of J/cm2, they advocated expressing and comparing laser dosage in joules per month. Our study used different wavelengths, output power, spot diameter, and exposure time than the others. Our energy density was chosen after pilot research with the study's wavelength and spot size. Our study applied high energy density every two days, which was 23.4 J/cm2 per day, like the prior investigations. 15, 20 BMMSCs were injected buccally once between central incisor roots. Previous research used MSCs more than once due to their short life cycle in host tissues (3 days).^22^, ^23^, ^24^

Amuk et al. ^17^ used 1x10^3^ MSCs to inhibit and repair orthodontic root resorption, which was similar to the number of cells used in our study but less than the number of cells used in other animal studies used (1x10^6^) cells. However, the periodontal and bone defects induced in these two studies^17^ were more significant than that influenced by the orthodontic movement.

Regarding relapse rate, we postulated LLLT-induced biostimulation of collagenase and gelatinase boosted relapse rate in the absence of retention, keeping collagen degradation and synthesis in an active state. The L group had the highest relapse rate during the 4-day study. Group L's recurrence rate decreased from 4 days to 4 weeks due to the laser's stimulatory effects on osteoclasts, osteoblasts, fibroblasts, and endothelial cells. ^11^ In other periods of the experiment, the relapse rate decreased due to a decrease in medial pressure from collagen degradation. Franzen et al. ^20^ and Kim et al. found similar results.^15^

MSCs sense mechanical impulses and respond accordingly.^23^, ^24^, ^25^ MSCs have a high capacity for osteogenic differentiation,^26^ and PDLSCs have key functions in periodontal and alveolar bone remodeling.^27^ Zhang et al. ^28^ found that PDLSC track markers increased after three days of orthodontic force administration. This explains our study's lowest 4-day relapse rate. Although removing retention means boosted relapse force, which is similar to OTM force, relapse did not arise over the four days due to the direct and indirect effect of BMMSCs. Injected BMMSCs elicit immunomodulatory effects and reduce inflammation at the injection site,^10^ promoting PDL and bone remodeling over the one-week retention period. This explains why the relapse rate was low early in the trial because the PDL and bone fight relapse.

Stem cell transplantation into the pressure location may accelerate OTM.^29^ This could occur by stimulation of PDLSC by transplanted MSCs or direct proliferation and differentiation potential and MSCs activity in PDL tissues, which accelerated OTM by accelerating osteoblast–osteoclast turnover.^30^ Stem cell activation in PDL activates inflammatory markers, including IL-11 and Cox-2, which govern osteoclast and osteoblast proliferation, differentiation, and bone remodeling.^31^, ^32^ These findings could explain the MSC group's increased relapse rates during other research periods.

For MSCs-L, the relapse rate was low for four days and four weeks but high for subsequent research periods. The 4-day results of this group match those of prior studies^27^, ^28^, ^29^, ^33^ on MSC's effects. The low relapse rate for the 4-week period indicates the effects of LLLT, as OTM has stopped or diminished during this time. Our results agreed with Fekrazad et al.,^34^ who found no synergy between MSC and LLLT.

Yoshida et al.^3^ found that tension-side BMD reduced during relapse. In our investigation, the LLLT group had the highest bone density in most periods. Franzen et al. ^20^ postulated that this was due to the promotion of osteogenesis by LLLT at tension sites, which balances osteoclast genesis and bone resorption at pressure and tension sites. Our study used more precise 3-D radiography than Franzen et al..^35^, ^36^ The MSCs group's bone density gradually increased over four days, but it was still lower than the C groups. MSC injections may have activated PDLSCs, which raised the pace of OTM that handled increasing bone resorption on the pressure side. MSCs increase the pressure side osteoclast count.^31^, ^32^

In most experiment intervals, the number of osteoclasts on the mesial side of the roots was higher than on the distal side in all groups. Many studies showed LLLT's significance in bone remodeling by enhancing osteoclast differentiation and activity. 37, 38 Mitochondria absorb light, which increases ATP generation and cell activity. ^39^ LLLT affects osteoclasts because of their high mitochondrial activity. The bone matrix releases a light-dose-dependent protein that induces osteoclast development. ^40^ This may explain the L group's increased osteoclast count. Cossetin et al. ^41^ reported that osteoclasts increased one week after LLLT force application. The discrepancy in magnitudes between our study's relapse force and the orthodontic force employed in the other study may explain why the L group's osteoclast count remained high for a week. The decrease in osteoclast count in the L group at 3 and 4 weeks may be due to LLLT maturing pre-osteoclasts in the periodontal ligament without prompting bone marrow cells to develop rapidly into new pre-osteoclasts. 13, 27

MSC transplantation promotes osteoclast differentiation and number. Wang et al.^43^ noted that compressive stress and MSCs synergized to induce osteoclast activity on the pressure side of the tooth and accelerate the orthodontic movement. Eggenhofer and colleagues ^30^ concluded that MSCs activated PDLSC by transplanting or by direct effect in PDL tissues accelerated OTM by accelerating bone remodeling through osteoblast–osteoclast turnover. Wang et al. ^42^ showed that osteoclasts increased considerably for ten days, consistent with our findings.

Abe et al. ^43^ reported MSCs suppressed osteoclast differentiation, while Ekizer et al. ^44^ found no difference in osteoclast quantity. The first study was conducted in vitro to examine MSCs on proteins that interfere with osteoclastic differentiation, whereas the second study analyzed the number of osteoclasts when compressive forces were reduced.

LLLT had a positive effect on the proliferation and differentiation of PDL cells, such as fibroblasts,^13^, ^46^ osteoblasts,^13^, ^47^ epithelial cells, and vascular cells. ^13^^.^ Liu et al. ^47^ found that LLLT increased PDL cell viability, decreased inflammatory markers, and increased bone formation 5–7 days after laser application.

The histological findings for the L group in our study showed a bio-stimulatory effect of LLLT on PDL cells, and these findings were in accordance with the studies mentioned above. However, the detected resorptive activity and disturbances in the PDL on the mesial side during the 2-week period in our study were attributed to the pressure exerted by relapsed tooth movement, which increased osteoclastic activity and was also stimulated by laser biostimulation.^13^, ^20^, ^36^, ^37^

During the four days of trial, the MSC group on the pressure side showed resorptive effects and restructuring of PDL fibers and blood vessels. Early resorption may be due to the synergistic action of pressure and MSCs on the PDL, which stimulated osteoclastic activity and increased osteoblast-osteoclast turnover.^30^ MSCs induce the production of PDLSCs, which facilitates the remodeling process by regulating osteoclast and osteoblast proliferation and differentiation^30^, ^31^, ^32^ and encouraging new bone and blood vessel creation.^42^, ^43^, ^44^ Ekizer et al. ^44^ found that injecting BMMSCs into the intermaxillary suture resulted in new bone formation indicated by increased osteoblasts number. They claimed the newly formed bone resulted from both the direct action of MSCs through osteogenic differentiation and indirectly by releasing growth factors. The novel element in the provided investigation was the relapse teeth movement pressure. Therefore, new bone formation in the MSCs group occurred with decreased PDL pressure.

Choi et al.^45^ found that LLLT enhanced bone regeneration by influencing the functionality as well as the survival and proliferation of adipose-derived stem cells. Furthermore, their effects appeared during the last periods of the experiment (28 and 56 days), which were per our study results. Mohaghegh et al. showed the same findings that within 21 days, LLLT and MSC showed promising results regarding new bone formation in the midpalate suture. ^48^ Kouhkheil et al.^49^ found that the simultaneous use of LLLT and BBMSCs showed a synergistic effect on infected wound healing. However, the present study differs from the above-mentioned studies ^(^34, 45, 48^)^ in that the present study tries to determine the histological changes during the relapse movement of the teeth, while the above-mentioned studies determined the histological changes during the retention phase.

When comparing the histology and radiographic findings of this investigation about root relapse, the histological events came first. Radiographically, the L group had the highest relapse rate, and histologically, PDL reorganized after four days. In the 1-week interval, the L group had the lowest radiographic relapse rate, showing that the restructured PDL findings in the 4-day period imply stability in relapse radiographically. Radiographs underestimate tooth and jaw development. ^50^ The MSC group had the highest radiographic recurrence rate at four days but the lowest histology relapse rate at one week.

The small sample size and the unmeasurable amount of force considered the main limitation in the presented study.

## 5 Conclusions

Based on radiographic and histological results, we conclude that LLLT could minimize relapse. Rats should use LLLT after two weeks of retention removal. The transfer of BMMSCs at the pressure side exhibited good effects on reconstructing PDL and lowering but not blocking relapse. These benefits were equivalent to those of LLLT, but they appeared 4 to days after retention removal. LLLT and BMMSC had stronger synergistic effects than group C; however, they were still modest and long-lasting after removing retention methods.

## Ethics approval and consent to participate

The experimental animal protocol was approved by the Institutional Animal Care and Use Committee (CU-IACUC) (Approval No. CUIF6518).

## Funding

This research received no specific grant from any funding agency in the public, commercial, or not-for-profit sectors.

## Data availability

Data supplied in this manuscript shall be supplied upon request.

## Conflict of interest

The authors declare no conflict of interest.

## CRediT authorship contribution statement

**Samer S. Othman:** Conceptualization. **Ali Saafan:** Conceptualization. **Mohammad M.F. Al-Halbosiy:** Methodology. **Iman Fathy:** Validation. **Mohamed Khursheed Alam:** Formal analysis. **Amr R. El-Beialy:** Formal analysis, Data curation. **Hanady S. Al-Shmgani:** Writing – original draft, Visualization. **Ghassan M. Sulaiman:** Writing – original draft, Visualization, Data curation.
